# Supplementary material for: Patterns of Intron Gain and Loss in Fungi
Source: PLoS Biol. 2004 Nov 30;2(12):e422. doi: 10.1371/journal.pbio.0020422 (PMC532390; doi:10.1371/journal.pbio.0020422)
Supplement: Table S1 — Also available at http://genes.mit.edu/NielsenEtAl/. (4.3 MB ZIP). [file pbio.0020422.st001.zip › NielsenEtAl/html/648.html]

AN1057.1.NCU09980.1.MG03077.1.FG09417.1


```
 CLUSTAL W (1.82) Multiple Sequence Alignments - Introns Inserted


Sequence 1: NCU09980.1	920 aa
Sequence 2: MG03077.1	820 aa
Sequence 3: FG09417.1	699 aa
Sequence 4: AN1057.1	774 aa
Alignment Length: 1053 aa
Number Identitical Residues: 188 aa
Alignment Score (without introns) 10832


MG03077.1 	-------~--------~---------------------~---------------------
NCU09980.1	MGRWSSD1LSEEEVLG2ARPKRESHRLGTVLRKKPQIS2IYGFTFRTYLFPSKWKRSFRT
FG09417.1 	-------~--------~---------------------~---------------------
AN1057.1  	-------~--------~---------------------~---------------------
          	                                                            

MG03077.1 	-----------------------------MMSAQLQQQAN--------------------
NCU09980.1	HPALQLLPILSVAFEAIPESDSAASQSPKMMSAQLQQQQQKQQQDQQQKQQKQQNQPQHQ
FG09417.1 	-----------------------------MVASELT------------------------
AN1057.1  	------------------------------------------------------------
          	                                                            

MG03077.1 	------------AAAAAAASRGEGVATPAASETQAIEAIAPGTESPT----LRNRAGDSE
NCU09980.1	LSQQQLHQSLPSRTGSGSVSANASIAPGAAGSQIPGAPAASNTQQPASFGQLRNRAAESQ
FG09417.1 	--------------ASAIAMRSTPVSSDESASSLP-RPLSP----------LQNRAASSR
AN1057.1  	--------------MQAQRHQGHDTASGVGP-------------------KLVNRLEASK
          	                .    .   :.  .                    .* **   *.

MG03077.1 	SPY~IQAHQDTPVAWQLLDKDAVALAKSQNKLIFMNIGFKACH-~---------------
NCU09980.1	SPY~VRVHAKTPVAWQLLNADTLKRAQAENKPIFMHIGFLADHH1CRLTTQDSFSNNAVA
FG09417.1 	SPY~IRDHAKDLVAWQLLDDEAVERSRKENKLIFLNIGYKACHF1CRLMSIETFSNPESA
AN1057.1  	SPY0VRAHRHNPVAWQLWDAESMELARRHNRLIFLSIGYSACHW1CHVMEKESFMSQEVA
          	*** :: * .  ***** : :::  :: .*: **: **: * *  .     .:  .   :

MG03077.1 	-----------------------------S1VNSAGGWPLNVFLTPELEPVFGGTYWPGP
NCU09980.1	AFLNSSFIPVIIDRDERPDLDTIYQNYSEA~VNATGGWPLNLFLTPDLYPIFGGTYWPGP
FG09417.1 	AVLNESFVPVIVDREERPDIEAVYMNYAQA~VYKVGGWPLNVFLTPNLEPVFGGTYWVGP
AN1057.1  	SILNESFIPIKVDREERPDVDDIYMNYVQA~TTGSGGWPLNVFLTPDLEPVFGGTYWPGP
          	:  ..:  .   . .. .. .    .  .: .   ******:****:* *:****** **

MG03077.1 	GRSTSSAVEDG----------------------EEPLDFLGILKKLQKVWTEQEAKCRKE
NCU09980.1	GTEHSLAAARGGASGVGGVAATPEASSINGGGEESYNDFLAIAKKIHKFWVEQEERCRRE
FG09417.1 	TGRRRHNGDST----------------------DEVLDSLTIFKKMRDTWNDQEARCRKE
AN1057.1  	NAASLLG--------------------------PETVSFIEILEKLRDVWQTQRQRCLES
          	                                  .  . : * :*::. *  *. :* ..

MG03077.1 	AQDIVLQLREFAAEGTMGVGNTEKVPSVATTGATVNISTGVAAPTTSTETPKKTVTASAS
NCU09980.1	AFEMLHKLQDFAQEGTFSGTPAEPVPVVAP------------------------VAAADV
FG09417.1 	ATEIVAQLKEFAAEGTLGTRSITAPSALGP----------LAGWGAPAPSNLSTTENRTM
AN1057.1  	AKEITKQLREFAEEGTH--------------------------------------TFQGD
          	* ::  :*::** ***                                            

MG03077.1 	ATDLDVDLDQLEEAYANISRSFDRVNGGFNLSPKFPTPPKLSFLLRLAHLPPEVGDIVGG
NCU09980.1	EAGADLDLDQLDEALDRIFKMFDPVDCGFG-TPKFPNPARLSFLLRLAQFPREVRDVVGD
FG09417.1 	IVSQELDLDQLEVAYRNIVSTFDLVHGGFGLAPKFVIPPKLTFLLGLLTAPGSVQDVVGY
AN1057.1  	QSDEDLDVELLEEAYQHFASRYDINNGGFSRAPKFPTPANLSFLLRLGIYPSAVTDIVGQ
          	  . ::*:: *: *  .:   :*  . **. :***  *..*:*** *   *  * *:** 

MG03077.1 	PEEIARATHMALATLRALRDGGLRDHIGAG-FHRYSVTADWSVPHFEKMIADNALLLGVY
NCU09980.1	-KEVENAASMARSTLRRIRDGGLRDHVGAG-FMRFSVTSDWSMPHFEKMVGENALLLGVY
FG09417.1 	-DECRHATKIALDTLRQIRDGALHDHIGATGFSRCSVTADWSIPNFEKLVIDNAQLLSLY
AN1057.1  	-EECENATAMAVSTLISMARGGIRDHIGHG-FARYSVTADWSLPHFEKMLYDQAQLLDVY
          	 .*  .*: :*  **  :  *.::**:*   * * ***:***:*:***:: ::* **.:*

MG03077.1 	LDAWLG--QAAKEGRAPTLEDEFADVVLELGDYLGNPGSEFGSSSTCQDSLLPTSEASDS
NCU09980.1	LDAWLGRVQSSAAETRLSLEDEFADVVIDLADYLTSPLIQFSGGG------FVTSEAADS
FG09417.1 	IDAWKA---SGGGE-----QGEFLDVVLELIDYLTTSPVTLPEGG------FASSEAADS
AN1057.1  	ADAFKIT-----------HNPEFLGAVYDLITYLTSAPIQSTTGG------FHSSEDADS
          	 **:               : ** ..* :*  ** ..      ..      : :** :**

MG03077.1 	YQRKSDKHMREGAFYLWTRREFDATVSNTEDGDLTNGKHDGDFYARVAAAYWNVKEHGNI
NCU09980.1	FYRKGDRHMREGAYYLWTRREFDDVVGPAGS-------------AEVAAAYWNVLEDGNI
FG09417.1 	YYRQGDNEKREGAYYVWTWREFKSVLDDIDH-----------HMSPILAAYWNVNKDGNV
AN1057.1  	LPTPNDTEKREGAFYVWTLKELTQVLGPRDAG--------------VCARHWGVLSDGNI
          	    .* . ****:*:** :*:  .:.    .              : * :*.* ..**:

MG03077.1 	PEEQDPHDEFINQNVLRVVK--TPAELSTSFGIAVDEVNQILAEARRKLRARRDSDRVRP
NCU09980.1	PRDQDPHDEFINQNVLCSVWGKDTQALSKQFGIPVNDVKKIIAKARERLRAHREQERPRP
FG09417.1 	KETNDPNDDFENQNILCVKT--TVEQLSSHFSTPVEKVREYIEKGKAALRKKREQERVRP
AN1057.1  	APENDPHDEFMDQNVLSIKV--TPSKLAKEFGLGEDEVVRIIKSGRQRLREYRDKNRVRP
          	   :**:*:* :**:*          *:. *.   :.* . : ..:  **  *:.:* **

MG03077.1 	EVDEKQVVAYNAMAMSALARAGVVLWSTGLDKHRGSAWMMCAKQAAIEMKGRLYDQETG-
NCU09980.1	ARDEKVVVGVNGMVISALARTAAVVR--ELDKTKSQKYLEAAQQAAAFIKENLWVQDGTQ
FG09417.1 	ELDDKIVAGWNGLVISALSKAASALR--TLKPEQSSRCKSAAERAAACIKERLWDADEK-
AN1057.1  	DLDDKIIVAWNGLAIGALAKCSVLFE--EIDSSKSAQCREAAAKAINFIKETLYDKATG-
          	  *:* :.. *.:.:.**:: .  .    :.  :.     .* :*   :*  *:      

MG03077.1 	--KLSRHWFRNKKSSTDALAEDYAFLIEALLDLYDATGDESAYLDWAKQLQD1KQIEMFY
NCU09980.1	SRKVLKRFWFNQPSDTRAFADDYAFLIEGLLDLYEAT-LEVKWLVWAKELQD1VQSELFY
FG09417.1 	---VLYRTWSGERGHT-AFADDYAYLIQGLLDLFELT-ENHHYLEFAETLQP1HSP----
AN1057.1  	--QLWRIYRDGSKGTTPGFAEDYAFLTSGLLDMYEAT-FDDSYLQFAEQLQR1YLNENFL
          	  .:      .. . * .:*:***:* ..***::: *  :  :* :*: **     .   

MG03077.1 	DRVAPSSQNLDSDAAKTKSGSGGFYSTAEEAP-DVILRLKDGMDTSQPSTNAVSASNLFR
NCU09980.1	D-----TPVVGSTPSLRHSYTGGFYSTEEATLSHTILRLKSGMDKSQPSTNAVSASNLFR
FG09417.1 	---------------------------------HVILRLKEGMDTSLPSTNAVSVANLFR
AN1057.1  	AY-----------AGSSPAGYYTTPSTSAPGSPATLLRLKTGTESAVPSVNGVIARNLLR
          	             ..   :      ::     . .:**** * :.: **.*.* . **:*

MG03077.1 	LALILDNLESSTDGTKTSRRHNYNTLARETIKAFEVEMLQYPFLFTGLLTSVVSARLGGQ
NCU09980.1	LGTILD-------------EKPFIRQAIETINAFEAEILQYPWLFVSLLAGVVTLRLG--
FG09417.1 	LASLLL-------------DEELTTKARQTINAFEIEVAQYPWLFPGLLGCVVTERLGN-
AN1057.1  	LSSILE-------------ENSYRVLARQTCQSFAVEILQHPFLFVGLLDVIVGLNTGTR
          	*. :*               .     * :* ::*  *: *:*:** .**  :*  . *  

MG03077.1 	-------AVFTDDKQRLGGEDASSVIAREFACKPRGGLRALCVKRKDTAPREVSDGVSGI
NCU09980.1	-----------VRETRVKVEQTAS--LRQYWKTPKAGANALLLGADTRG-----------
FG09417.1 	------------------------------------------------------------
AN1057.1  	NITAVVAADSSLSTSTEGGTPTSAWDGLVKRIRAEAGPTLSTSSNTVAALVDVRNSSQGV
          	. ::  ::  :          :::         . ..           .  .  .. .. 

MG03077.1 	TGG------------IEQLKTGEH----------------
NCU09980.1	----------------------------------------
FG09417.1 	----------------------------------------
AN1057.1  	STGNKSSWLRRRNPLFKDLKAGDKNYLLICEEGRCRTVNL
          	: ...::     ..  .. .:.. .    .... . : .
```
